# “Delirium Day”: a nationwide point prevalence study of delirium in older hospitalized patients using an easy standardized diagnostic tool

**DOI:** 10.1186/s12916-016-0649-8

**Published:** 2016-07-18

**Authors:** Giuseppe Bellelli, Alessandro Morandi, Simona G. Di Santo, Andrea Mazzone, Antonio Cherubini, Enrico Mossello, Mario Bo, Angelo Bianchetti, Renzo Rozzini, Ermellina Zanetti, Massimo Musicco, Alberto Ferrari, Nicola Ferrara, Marco Trabucchi, on behalf of the Italian Study Group on Delirium (ISGoD), Stefano Boffelli, Fabio Di Stefano, Francesco De Filippi, Fabio Guerini, Erik Bertoletti, Albert March, Alessandro Margiotta, Patrizia Mecocci, Desireè Addesi, Fausto Fantò, Gianluca Isaia, Babette Dijik, Paola Porrino, Antonino Maria Cotroneo, Giovanni Galli, Amalia Cecilia Bruni, Bruno Bernardini, Carla Corsini, Annachiara Cagnin, Amedeo Zurlo, Giuseppe Barbagallo, Maria Lia Lunardelli, Emilio Martini, Giuseppe Battaglia, Raffaele Latella, Donatella Petritola, Elena Sinforiani, Alberto Cester, Marino Formilan, Pasqualina Carbone, Ildebrando Appollonio, Diletta Cereda, Lucio Tremolizzo, Edo Bottacchi, Lucio Lucchetti, Claudio Mariani, Piero Rapazzini, Giuseppe Romanelli, Alessandra Marengoni, Giovanni Zuliani, Lara Bianchi, Teresa Suardi, Ettore Muti, Renato Bottura, Giovanni Sgrò, Antonella Mandas, Luca Serchisu, Patrizia Crippa, Claudio Ivaldi, Andrea Ungar, Daniele Villani, Clara Raimondi, Chiara Mussi, Giancarlo Isaia, Giuseppe Provenzano, Daniela Mari, Patrizio Odetti, Fiammetta Monacelli, Raffaele Antonelli Incalzi, Alice Pluderi, Claudio Bellamoli, Luciano Terranova, Elio Scarpini, Ferdinando D’Amico, Maria Chiara Cavallini, Gianbattista Guerrini, Anna Maria Scotuzzi, Antonino Chiarello, Alberto Pilotto, Sara Tognini, Giuseppina Dell’Aquila, Gabriele Toigo, Giuliano Ceschia, Maristella Piccinini, Andrea Fabbo, Marco Zoli, Paola Forti, Christian Wenter, Giorgio Basile, Anna Lasagni, Alessandro Padovani, Luca Rozzini, Maria Cottino, Silvia Vitali, Gabriele Tripi, Stefano Avanzi, Giorgio Annoni, Giovanni Ruotolo, Federica Boschi, Paolo Bonino, Niccolò Marchionni, Maria C. Cavallini, Sara Fascendini, Gabriele Noro, Renato Turco, Maria C. Ubezio, Carlo Serrati, Maria Infante, Simona Gentile, Luigi M. Pernigotti, Carlo A. Biagini, Enzo Canonico, Pietro Bonati, Pietro Gareri, Paolo Caffarra, Arcangelo Ceretti, Rosanna Castiglia, Carlo Gabelli, Mario Lo Storto, Paolo Putzu, Giuseppe Bellelli, Alessandro Morandi, Simona Di Santo, Andrea Mazzone, Renzo Rozzini, Ermellina Zanetti, Angelo Bianchetti, Mario Bo, Enrico Mossello, Antonio Cherubini, Nicola Ferrara, Alberto Ferrari, Massimo Musicco, Marco Trabucchi

**Affiliations:** School of Medicine and Surgery, University of Milano-Bicocca, Milano, Italy; Geriatric Unit, San Gerardo University Hospital, Monza, Italy; Geriatric Research Group, Brescia, Italy; Department of Rehabilitation and Aged Care “Fondazione Camplani” Hospital, Cremona, Italy; Department of Clinical and Behavioral Neurology, Neuropsychiatry Laboratory, IRCCS Foundation S Lucia, Roma, Italy; Redaelli Geriatric Institute, Milan, Italy; Geriatrics and Geriatric Emergency Care, IRCCS-INRCA, Ancona, Italy; Research Unit of Medicine of Ageing, Department of Experimental and Clinical Medicine, University of Florence and Azienda Ospedaliero-Universitaria Careggi, Firenze, Italy; Section of Geriatrics, Città della Salute e della Scienza – Molinette, Torino, Italy; Medicine and Rehabilitation Department, Istituto Clinico S. Anna, Brescia, Italy; Department of Geriatric and Internal Medicine, Poliambulanza Hospital, Brescia, Italy; Institute of Biomedical Technologies, National Research Council, Segrate (Milan), Italy; Italian Society of Neurology for Dementia (SINDEM), Siena, Italy; Geriatric Unit, Department of Neuromotor Physiology, ASMN Hospital, Reggio Emilia, Italy; Italian Society of Hospital and Community Geriatrics (SIGOT), Roma, Italy; Department of Translational Medical Sciences, Federico II University of Naples, Naples, Italy; Salvatore Maugeri Foundation, IRCCS, Scientific Institute of Telese, Telese Terme (BN), Italy; Italian Society of Gerontology and Geriatrics (SIGG), Florence, Italy; Tor Vergata, Rome University, Rome, Italy; Italian Psychogeriatric Association (AIP), Brescia, Italy

**Keywords:** Delirium, Prevalence, Hospital, Multicenter, 4AT

## Abstract

**Background:**

To date, delirium prevalence in adult acute hospital populations has been estimated generally from pooled findings of single-center studies and/or among specific patient populations. Furthermore, the number of participants in these studies has not exceeded a few hundred. To overcome these limitations, we have determined, in a multicenter study, the prevalence of delirium over a single day among a large population of patients admitted to acute and rehabilitation hospital wards in Italy.

**Methods:**

This is a point prevalence study (called “Delirium Day”) including 1867 older patients (aged 65 years or more) across 108 acute and 12 rehabilitation wards in Italian hospitals. Delirium was assessed on the same day in all patients using the 4AT, a validated and briefly administered tool which does not require training. We also collected data regarding motoric subtypes of delirium, functional and nutritional status, dementia, comorbidity, medications, feeding tubes, peripheral venous and urinary catheters, and physical restraints.

**Results:**

The mean sample age was 82.0 ± 7.5 years (58 % female). Overall, 429 patients (22.9 %) had delirium. Hypoactive was the commonest subtype (132/344 patients, 38.5 %), followed by mixed, hyperactive, and nonmotoric delirium. The prevalence was highest in Neurology (28.5 %) and Geriatrics (24.7 %), lowest in Rehabilitation (14.0 %), and intermediate in Orthopedic (20.6 %) and Internal Medicine wards (21.4 %). In a multivariable logistic regression, age (odds ratio [OR] 1.03, 95 % confidence interval [CI] 1.01–1.05), Activities of Daily Living dependence (OR 1.19, 95 % CI 1.12–1.27), dementia (OR 3.25, 95 % CI 2.41–4.38), malnutrition (OR 2.01, 95 % CI 1.29–3.14), and use of antipsychotics (OR 2.03, 95 % CI 1.45–2.82), feeding tubes (OR 2.51, 95 % CI 1.11–5.66), peripheral venous catheters (OR 1.41, 95 % CI 1.06–1.87), urinary catheters (OR 1.73, 95 % CI 1.30–2.29), and physical restraints (OR 1.84, 95 % CI 1.40–2.40) were associated with delirium. Admission to Neurology wards was also associated with delirium (OR 2.00, 95 % CI 1.29–3.14), while admission to other settings was not.

**Conclusions:**

Delirium occurred in more than one out of five patients in acute and rehabilitation hospital wards. Prevalence was highest in Neurology and lowest in Rehabilitation divisions. The “Delirium Day” project might become a useful method to assess delirium across hospital settings and a benchmarking platform for future surveys.

**Electronic supplementary material:**

The online version of this article (doi:10.1186/s12916-016-0649-8) contains supplementary material, which is available to authorized users.

## Background

Delirium is an acute and fluctuating disorder of attention and cognitive functioning, which is almost always triggered by underlying medical causes and is often accompanied by abnormal arousal and perceptual disturbances [1]. Delirium is associated with many adverse clinical outcomes, including reduction of functional independence, worsening of cognitive performance, and increased mortality [2–4]. Importantly, the mortality risk is associated with delirium per se, independent of the associated medical conditions, and is strongly related to delirium duration [2, 5]. Delirium is also associated with increased costs of care, with more than US$ 164 billion per year expended in the USA and more than 182 billion Euros per year in Europe [6, 7]. Furthermore, delirium causes higher distress for patients, caregivers, and healthcare professionals [8, 9].

However, despite this burden, delirium often goes unrecognized, and its detection is still grossly inadequate in clinical practice [10–12]. Physicians often do not assess a patient’s cognition [13, 14], and even if they do, they often fail to recognize the importance of delirium as the interface between mental and physical health [12, 15]. This is particularly relevant because the inability to detect delirium implies an increased risk of poor outcomes for patients [16]. Conversely, increased knowledge concerning delirium might allow the staff to implement preventive interventions [17] which have been consistently shown to reduce the incidence of delirium in hospitalized patients [18, 19] and to ultimately improve their outcomes [20].

A recent review of 35 studies published between 1990 and 2012 reported a prevalence of delirium ranging from 11–42 % in acute hospitals and from 14–18 % in long-term care and post-acute rehabilitation [4, 21, 22]. However, most of these estimates refer to only a few hundred patients, are based on pooled results from different studies carried out in single wards or hospitals [23–26], and are limited by some methodological heterogeneity. Furthermore, all of them have been performed using diagnostic tools that require preliminary training and/or longstanding experience to be used efficiently [14, 27, 28], with consequent poor transferability of results to clinical practice. To date, only one study assessed delirium across multiple wards of a tertiary hospital within a 24-hour period, finding an average prevalence of 20 %, but, again, the diagnostic procedures to assess delirium were strongly dependent on the researcher’s expertise in delirium [26].

We report here the first nationwide point prevalence study to assess delirium in older (i.e., aged 65 years or more) patients admitted to acute and rehabilitation hospital wards, using a standardized tool that requires neither longstanding experience nor a preliminary training of the assessors [29]. This study was a part of a larger surveillance project, called “Delirium Day,” which involved not only acute and rehabilitation hospital wards but also nursing homes, and was widely distributed across Italy.

## Methods

During the 14th National Congress of the Italian Association of Psychogeriatrics (AIP), held in Florence in 2014, an interdisciplinary group of clinicians and researchers belonging to four Italian scientific associations (the AIP, the Italian Society of Gerontology and Geriatrics [SIGG], the Italian Society of Hospital and Community Geriatrics [SIGOT], and the Italian Society of Neurology for Dementia [SINDEM]) discussed the current state of delirium knowledge among healthcare operators in Italy and shared ideas on advancing the best-care practices in this field. The Delirium Day was the result of this collaborative effort. This initiative was conceived both as a method to assess delirium prevalence in various settings of care and as an innovative project to assess delirium to disseminate culture and awareness of the issue among healthcare staff.

### Brief description of the Italian hospital care system

The hospital care in Italy is delivered by more than 600 public and more than 500 private hospitals, especially not-for-profit institutions, which provide both outpatient and inpatient services. The number of hospital beds is approximately 330,000, i.e., less than 3.7 per 1000 inhabitants and there are about 1.78 hospital doctors per 1000 resident population [30]. The facilities participating in this study are all part of the Italian hospital care system.

### Study design

The invitation to participate in this multicenter “point prevalence” study was sent via email to the members of the four scientific associations (globally about 5000), with publication on their websites. Each member was also encouraged to advertise the project and to invite participation by other facilities. Members who voluntarily agreed to participate were invited to send a confirmation email to the following address: deliriumday2015@gmail.com by 15 August 2015. No incentives were offered to participants.

### Subjects and study protocol

September 30, 2015 was the day chosen for the study (index day). All patients admitted to the participating centers from 00:00 to 23:59 of the index day were considered potentially eligible if they were aged 65 years and older, were native Italian speakers, and if they or a proxy provided a written informed consent. Exclusion criteria were coma, aphasia, and end-of-life status, as defined by clinical judgment.

The assessment of the eligible patients was performed by the attending physicians with the following two-step approach.

#### Step 1 (mandatory for all patients)

- *Formal cognitive assessment:* physicians assessed the eligible patients using the Assessment test for delirium and cognitive impairment (4AT) [29]. The 4AT has recently been validated for the assessment of delirium in patients admitted to acute and rehabilitation hospital wards, showing a sensitivity of 89.7 % and a specificity of 84.1 % for delirium. Its administration is brief (generally less than 2 minutes), and it requires no special training, making delirium assessment feasible by untrained physicians or nurses. A score of 0 suggests neither delirium nor cognitive impairment; scores between 1 and 3 suggest possible general cognitive impairment (that is, corresponding to moderate to severe impairment on standalone dementia screening tools), while a score of 4 or above suggests likely delirium, based on the performance of the 4AT in the original validation study [29]. Thus, in the present study, delirium is defined as a score of 4 or more on the 4AT instrument. The 4AT form is shown in Additional file 1: Appendix A.
- *Clinical assessment:* For all patients a comprehensive sociodemographic and medical history was collected, including date of hospital admission and functional status prior to admission, using the Activities of Daily Living (ADL) score [31] and the Charlson comorbidity index [32]. Patients were deemed to have dementia if they had documented diagnosis in the medical record and/or were prescribed acetylcholinesterase inhibitors (AChE-I) or memantine prior to admission. Their nutritional status was evaluated referring to the time of assessment and classified according to clinical judgment of the attending physician, as “well nourished,” “at risk of malnutrition,” or “malnourished.” The total number of medications taken by each patient on the index day and the use of specific pharmacological classes (i.e., antihypertensives, antiplatelets, antiarrhythmics, statins/lipid lowering drugs, antidiabetics, antiulcer drugs, antibiotics, benzodiazepines, antipsychotics, antidepressants, antiepileptics, and AChE-I/memantine) were also recorded, together with the use of feeding tubes (i.e., nasogastric tube [NT] or percutaneous endoscopic gastrostomy [PEG] tubes), peripheral venous catheters, urinary catheters, and physical restraints (vests, wrists, inguinal restraints, and bedrails).

#### Step 2 (for patients scoring ≥4/12 on 4AT)

*Motor subtype of delirium:* this was measured using the Delirium Motor Subtype Scale (DMSS) [33, 34]. The DMSS is a scale using 11 motor items derived from items used in previous motor subtyping methods but with relative specificity for delirium and demonstrated correlation with objective measures of motor behavior, including electronic motion analysis [35]. It can be rated by any healthcare professional who is familiar with patient behavior, and it can be used to rate the previous 24 hours or more. Each of the 11 symptoms (4 hyperactive and 7 hypoactive features) is rated as present or absent where at least 2 symptoms must be present from either the hyperactive or hypoactive list to meet subtype criteria. Patients meeting both hyperactive and hypoactive criteria were deemed “mixed subtype,” while those meeting neither criterion were deemed “no subtype” [34].

### Data collection and ethical procedures

An electronic case report form (e-CRF) was created to collect clinical data, and each center was provided with a username and a password. After accessing the e-CRF, each clinician was asked to indicate the number of eligible patients and the number of those who accepted to participate. Then, an automatic message allowed the clinician to complete the data collection in the e-CRF. It was not possible to submit the data form without the mandatory clinical data. The submission was possible only after clicking on the “Finish survey” button when at least the mandatory assessment was terminated. All transcripts were anonymized, and none of the participants was allowed to see the data of patients from other centers or to identify their names.

The Ethical Committee of the IRCCS Fondazione Santa Lucia, Rome (Prot CE/PROG.500) approved the study protocol. Informed consent was obtained from all participants or from their next of kin when the participants were not capable of giving informed consent themselves because of delirium or severe cognitive impairment. Those who declined to participate in the study were excluded.

### Statistical analysis

The descriptive analysis for quantitative variables was based on calculation of the mean and standard deviation (SD) for parametric distribution or median and interquartile range (IQR) for nonparametric distributions, while qualitative variables were reported as frequencies and percentages. Comparisons between groups were performed using the one-way ANOVA or *t* test and the Kruskal-Wallis test or Mann-Whitney U test for normally and abnormally distributed data, respectively. Post hoc analyses were performed with the Tukey test or Dunn test when appropriate. The categorical variables were compared between groups using the chi-square test, and Bonferroni corrections were used for pairwise comparisons. We used univariate logistic regression analysis to evaluate the association of variables with delirium. Variables found to be statistically significant in the univariate analysis were included in a multiple age- and gender-adjusted logistic regression model in order to determine the factors independently associated with delirium. The level of significance was established as 95 % (*p* < 0.05). All analyses were performed using SPSS 22.0 (SPSS Inc., Chicago, IL, USA).

## Results

**Fig. 1:**
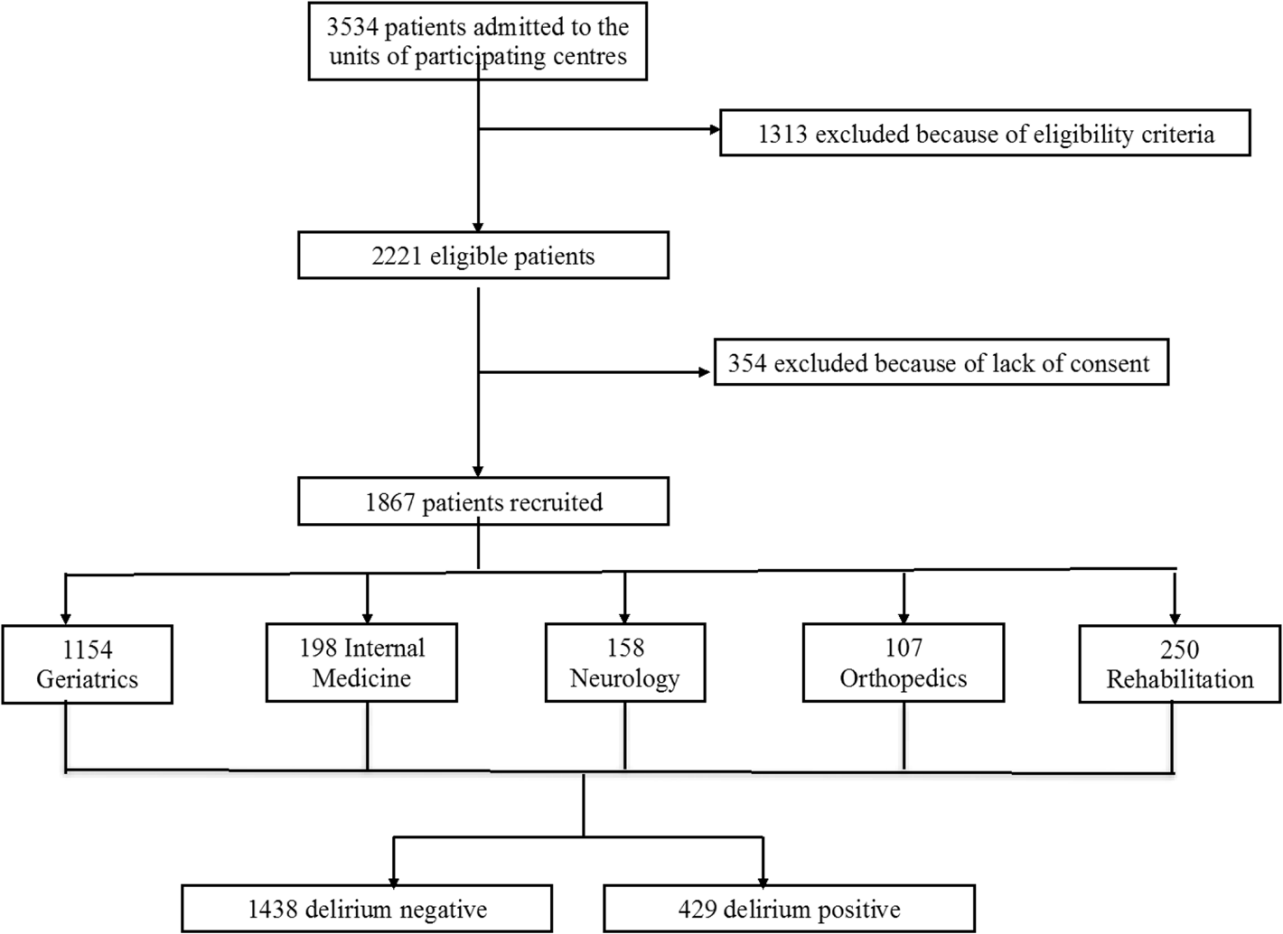
Disposition of participants in the study

A total of 161 centers responded to the invitation letter by sending a confirmation email; of these, 37 centers were excluded because they did not collect data on the index day, 2 because they recruited only one patient per center, and 2 because they included patients aged less than 65 years. Overall, 108 acute and 12 rehabilitation hospital wards were involved in the study, of which 60 were located in Northern Italy (30 Geriatrics, 11 Neurology, 9 Rehabilitation, 5 Orthopedics, and 5 Internal Medicine divisions), 40 in Central Italy (24 Geriatrics, 5 Internal Medicine, 5 Neurology, 4 Orthopedics, and 2 Rehabilitation divisions), and 20 in Southern Italy (8 Geriatrics, 7 Internal Medicine, 3 Orthopedics, 1 Neurology, and 1 Rehabilitation division). On the study day, 2221 patients were eligible. Of these, 354 did not consent to participate, leaving a sample of 1867 patients included in the study (Fig. 1). Of these, 1154 patients were from acute Geriatrics, 198 from Internal Medicine, 158 from Neurology, 107 from Orthopedics, and 250 from Rehabilitation wards. The mean age of the whole sample was 82.0 ± 7.5 years, and 58 % were female. Four hundred twenty-nine patients (22.9 %) had delirium, and of those the motoric subtype was characterized in 275 (64.1 %): hypoactive in 106 patients (38.5 %), mixed in 75 (27.3 %), hyperactive in 59 (21.5 %), and nonmotoric in 35 (12.7 %), according to DMSS scores.

Table 1 shows the demographic, cognitive, functional, nutritional, and clinical characteristics of subjects with and without delirium. Those with delirium were older and less educated than those without. Moreover, they were also more dependent in functional status, more often malnourished or at risk of malnutrition, and more frequently affected by dementia. There was no difference in regard to medications taken by patients with and without delirium, except for antihypertensives, statins/lipid lowering drugs, antiulcer drugs, and antibiotics, which were more frequently present in nondelirious patients, and for antipsychotics, antidepressants, and AChE-I/memantine, which were more frequently present in delirious patients. Finally, NTs/PEG tubes, peripheral venous and urinary catheters, and physical restraints were more prevalent among patients with delirium than in those without.

**Table 1:** Participant characteristics according to diagnosis of delirium

	Delirium (*N* = 429, 22.9 %)	No delirium (*N* = 1438, 77.1 %)	*p* value
Age, years	84.8 ± 6.7	81.1 ± 7.6	< .001
Female gender, *n* (%)	249 (58.0)	833 (57.9)	1.000
Education, years	6.2 ± 3.6	6.8 ± 3.8	.006
ADL before admission, median score (IQR)	1 (0–4)	5 (2–6)	< .001
0 functions spared	186 (43.4)	155 (10.8)	
1 function spared	66 (15.4)	174 (12.1)	< .001
2 to 5 functions spared	118 (27.5)	471 (33.8)	
6 functions spared	59 (13.8)	638 (44.4)	
Nutritional status, *n* (%)			
Well nourished	156 (36.4)	949 (66.0)	< .001
At risk of malnutrition	215 (50.1)	414 (28.8)	
Malnourished	58 (13.5)	75 (5.2)	
Charlson index (excluding dementia), median score (IQR)	2 (1–4)	2 (1–4)	.87
Dementia, *n* (%)	227 (52.9)	222 (15.4)	< .001
No. of drugs, on admission, median score (IQR)	5 (4–-7)	5 (3–7)	.56
Diuretics, *n* (%)	202 (47.1)	727 (50.6)	.22
Antihypertensive drugs, *n* (%)	241 (56.2)	939 (65.3)	.001
Antiplatelet drugs, *n* (%)	174 (49.6)	612 (42.6)	.47
Antiarrhythmic drugs, *n* (%)	39 (9.1)	173 (12.0)	.10
Statins/lipid lowering drugs, *n* (%)	45 (10.5)	297 (20.7)	< .001
Antidiabetics (including insulin), *n* (%)	72 (16.8)	284 (19.7)	.18
Antiulcer drugs, *n* (%)	270 (62.9)	980 (68.2)	.04
Antibiotics, *n* (%)	186 (43.4)	433 (30.1)	< .001
Benzodiazepines, *n* (%)	93 (21.7)	360 (25.0)	.16
Antipsychotics, *n* (%)	107 (28.3)	119 (9.1)	< .001
Antidepressants, *n* (%)	112 (26.1)	278 (19.3)	.003
Antiepileptics, *n* (%)	32 (7.5)	93 (6.5)	.51
AChE-I/memantine, *n* (%)	15 (3.5)	24 (1.7)	.03
Feeding tubes (NT or PEG), *n* (%)	20 (4.7)	20 (1.4)	< .001
Venous catheter, *n* (%)	244 (56.9)	694 (42.0)	< .001
Urinary catheter, *n* (%)	199 (46.4)	340 (23.6)	< .001
Physical restraints, *n* (%)	268 (62.5)	427 (29.7)	< .001

Data are expressed as mean + SD unless otherwise specified; *IQR* interquartile range, *p* value: significance at one-way ANOVA or *t* test and at Kruskal-Wallis test or Mann-Whitney U test for normally and abnormally distributed data, respectively

*ADL* Activities of Daily Living score, *AChE-I* acetylcholinesterase inhibitors, *NT* nasogastric tube, *PEG* percutaneous endoscopic gastrostomy

Table 2 shows the characteristics of patients according to the different settings. Those in Geriatrics wards were the oldest and also the most disabled and malnourished. Patients in Neurology wards were the youngest, the least malnourished, and the most frequently affected by dementia; they also were more often treated with benzodiazepines, antipsychotics, antidepressants, antiepileptics, and AChE-I/memantine, and least often with diuretics and antiplatelet medications. Patients in Orthopedic wards had the highest prevalence of peripheral venous and urinary catheter use, those in Internal Medicine wards had the highest antibiotic use, and those in Rehabilitation wards were the most educated and had the lowest frequency of peripheral venous and urinary catheters.

**Table 2:** Participant characteristics according to settings of admission

	Rehabilitation (*N* = 250)	Geriatrics (*N* = 1154)	Orthopedics (*N* = 107)	Neurology (*N* = 158)	Int. Medicine (*N* = 198)	*p* value
Age, years	80.4 ± 7.7^b,d^	83.2 ± 7.1 ^a,c,d,e^	80.8 ± 8.4 ^b,d^	77.7 ± 7.2 ^a,b,c,e^	80.7 ± 7.7 ^b,d^	<0.001
Female gender, *n* (%)	153 (61.2)	679 (58.8)	72 (67.3)^e^	81 (51.3)	97 (49.0)^c^	0.005
Education, years	7.3 ± 3.7^b,e^	6.5 ± 3.8 ^a^	7.2 ± 4.5	7.0 ± 3.7	6.9 ± 3.4^a^	0.004
ADL before admission, mean score	5 (1–6)^b^	4 (1–6)^a,c,d,e^	5 (3–6)^b^	5 (2–6)^b^	5 (2–6)^b^	<0.001
0 functions spared, *n* (%)	32 (12.8)	255 (22.1)	8 (7.5)	22 (13.9)	24 (12.1)	
1 function spared, *n* (%)	34 (13.6)	156 (13.5)	9 (8.4)	16 (10.1)	25 (12.6)	<0.001
2 to 5 functions spared, *n* (%)	92 (36.8)	349 (30.2)	41 (38.3)	45 (28.5)	62 (31.3)	
6 functions spared, *n* (%)	92 (36.8)	394 (34.1)	49 (45.8)	75 (47.5)	87 (43.9)	
Nutritional status, *n* (%)						
Well nourished	171 (68.4)^b,d^	601 (52.1)^a,d,e^	61 (57.0)^d^	130 (82.3)^a,b,c^	142 (71.7)^b^	<0.001
At risk of malnutrition	60 (24.0)^a^	457 (39.6)^b,d,e^	37 (34.6)^d^	27 (17.1)^b,c^	48 (24.2)^b^	
Malnourished	19 (7.6)^d^	96 (8.3)^d^	9 (8.4)^d^	1 (0.6)^a,b,c^	8 (4.0)	
Charlson index (excluding dementia)	1 (0–3) ^b,e^	3 (1–5) ^a,c,d^	1 (0–3) ^b,e^	2 (1–3)^b,e^	3 (1–4) ^a,c,d^	<0.001
Dementia, *n* (%)	50 (20.0)^d^	304 (26.3)^d^	18 (16.8)	60 (38.0)^a,b,e^	33 (16.7) ^d^	<0.001
No. drugs, on admission	5 (3–6)^e^	5 (4–7)^c^	4 (3–6)^b^	5 (3–6)^e^	6 (4–7)^a,d^	<0.001
Diuretics, *n* (%)	122 (48.8)^d^	624 (54.1)^c,e^	36 (33.6)^b^	80 (31.6)^a,b,e^	97 (49.0)^d^	<0.001
Antihypertensive, *n* (%)	157 (62.8)	723 (62.7)	62 (57.9)	99 (62.7)	139 (70.2)	0.228
Antiplatelet drugs, *n* (%)	93 (37.2)^d^	473 (41.0)^d^	37 (34.6)^d^	80 (31.6)^a,b,c,e^	97 (49.0)^d^	<0.001
Antiarrhythmic drugs, *n* (%)	27 (10.8)	139 (12.0)	7 (6.5)	11 (7.0)	28 (14.1)	0.106
Statins/lipid lowering drugs, *n* (%)	56 (22.4)	183 (15.9)^d^	15 (14.0)	41 (25.9)^b^	47 (23.7)	0.001
Antidiabetics (including insulin), *n* (%)	34 (13.6)	227 (19.7)	18 (16.8)	30 (19.0)	47 (23.7)	0.082
Antiulcer drugs, *n* (%)	166 (66.4)	783 (67.9)^d^	74 (69.2)	87 (55.1)^b,e^	140 (70.7)^d^	0.017
Antibiotics, *n* (%)	26 (10.4)^b,c,e^	448 (38.8)^a,d^	38 (35.5)^a,d^	22 (13.9)^b,c,e^	85 (42.9) ^a,d^	<0.001
Benzodiazepines, *n* (%)	74 (29.6)	259 (22.4)	25 (23.4)	51 (32.3)	44 (22.2)	0.018
Antipsychotics, *n* (%)	29 (11.6)^d^	163 (14.1)^d^	10 (9.3)^d^	42 (26.6)^a,b,c,e^	19 (9.6)^d^	<0.001
Antidepressants, *n* (%)	60 (24.0)	226 (19.6)^d^	22 (20.6)	50 (31.6)^b,e^	32 (12.6)^d^	0.003
Antiepileptics, *n* (%)	24 (9.6)^c,d^	61 (5.3)^d^	0^a,d^	34 (21.5)^a,b,c,e^	6 (3.0)^d^	<0.001
AChE-I/memantine, *n* (%)	4 (1.6)	22 (1.9)^d^	1 (0.9)	10 (6.3)^b^	1 (1.0)	0.003
Feeding tubes (NT or PEG), *n* (%)	3 (1.2)	23 (2.0)	4 (3.7)	5 (3.2)	5 (2.5)	0.492
Venous catheter, *n* (%)	26 (10.4)^b,c,d,e^	577 (50.0)^a,c,d,e^	75 (70.1)^a,b,d^	34 (21.5)^a,b,c,e^	136 (68.7) ^a,b,d^	<0.001
Urinary catheter, *n* (%)	28 (11.2)^b,c,d,e^	364 (31.5)^a,c^	66 (61.7)^a,b,d,e^	35 (22.2)^a,c^	46 (23.2)^a,c^	<0.001
Physical restraints, *n* (%)	68 (27.2)^b^	465 (40.3)^a^	44 (41.1)	52 (32.9)	62 (31.1)	<0.001

Data are expressed as mean + SD unless otherwise specified; *IQR* interquartile range, *ADL* Activities of Daily Living score, *AChE-I* acetylcholinesterase inhibitors, NT nasogastric tube, *PEG* percutaneous endoscopic gastrostomy

*p* denotes significance on ANOVA for continuous or chi-square for categorical variables. Where significant group effects were detected, either Turkey or Dunn or Bonferroni tests indicated significant post hoc differences between individual groups, as follows:

^a^Significant difference with Rehabilitation group

^b^Significant difference with Geriatrics group

^c^Significant difference with Orthopedics group

^d^Significant difference with Neurology group

^e^Significant difference with Internal Medicine group

**Fig. 2:**
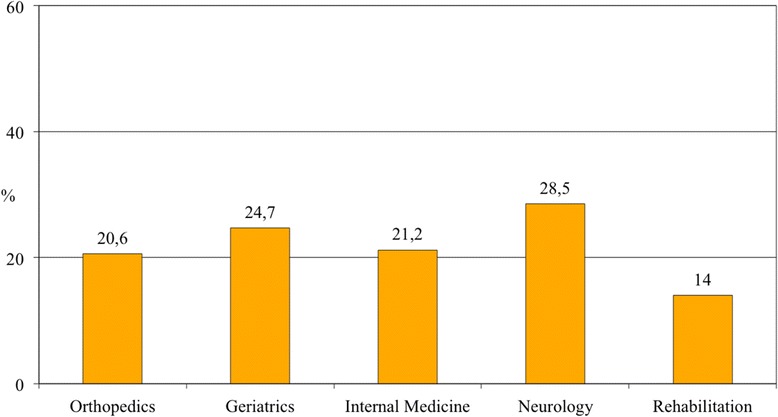
Prevalence of delirium according to different settings

The prevalence of delirium was highest in the Neurology (28.5 %; 45/158) and Geriatrics wards (24.7 %; 285/1154), lowest in Rehabilitation (14.0 %; 35/250), and intermediate in Orthopedic (20.6 %; 22/107) and Internal Medicine wards (21.2 %; 42/198) (Fig. 2). The overall delirium prevalence in acute hospital wards, excluding Rehabilitation, was 24.4 % (394/1617).

In the multivariable logistic regression analysis (Table 3) the following variables were significantly associated with delirium: age (OR 1.03, 95 % CI 1.01–1.05), ADL dependence (OR 1.19, 95 % CI 1.12–1.27), dementia (OR 3.25, 95 % CI 2.41–4.38), malnutrition (OR 2.01, 95 % CI 1.29–3.14), antipsychotics (OR 2.03, 95 % CI 1.45–2.82), feeding tubes (OR 2.51, 95 % CI 1.11–5.66), peripheral venous catheters (OR 1.41, 95 % CI 1.06–1.87), urinary catheters (OR 1.73, 95 % CI 1.30–2.29), and physical restraints (OR 1.84, 95 % CI 1.40–2.40). Moreover, taking admission to Rehabilitation as a reference category, being admitted to Neurology wards remained independently associated with delirium occurrence (OR 2.00, 95 % CI 1.10–3.64), while admission to other settings did not.

**Table 3:** Univariate and multivariate analyses of factors associated with delirium

		Univariate analysis			Multivariate analysis		
	Number	OR	95 % CI	*p* value	OR	95 % CI	*p* value
Age		1.07	1.05–1.09	<.001	1.03	1.01–1.05	.006
Female sex	180/785	.99	.80–1.24	.97	--	--	--
Education, years		.96	.93–.99	.008	1.00	.97–1.04	.77
Rehabilitation wards	35/250	Ref			Ref		
Geriatrics wards	285/1154	2.02	1.38–2.95	<.001	1.15	.72–1.83	.56
Orthopedic surgery wards	22/107	1.59	.88–2.87	0.12	--	--	--
Neurology wards	45/158	2.45	1.49–4.02	<.001	2.00	1.10–3.64	.02
Internal Medicine wards	42/198	1.65	1.01–2.71	.05	1.40	.77–2.57	.27
ADL (functions lost)		1.45	1.38–1.53	<.001	1.19	1.12–1.27	<.001
Dementia (yes/no)	243/465	7.16	5.64–9.08	<.001	3.25	2.41–4.38	<.001
Malnutrition (yes/no)	58/133	2.84	1.98–4.08	<.001	2.01	1.29–3.14	.002
Antihypertensives (yes/no)	241/1180	.68	.55–.85	.001	.84	.64–1.09	.18
Statins/lipid lowering (yes/no)	45/342	.45	0.32–0.63	<.001	.79	.53–1.17	.23
Antibiotics (yes/no)	186/619	1.78	1.42–2.22	<.001	1.24	0.93–1.64	.14
Antipsychotics (yes/no)	132/263	4.43	3.38–5.82	<.001	2.03	1.45–2.82	<.001
Antidepressants (yes/no)	112/390	1.47	1.15–1.90	.003	1.03	.76–1.41	.83
Antiulcer drugs (yes/no)	270/1250	.79	.63–.99	.04	.86	.65–1.13	.27
AChE-I/memantine (yes/no)	15/39	2.14	1.11–4.11	.03	.91	.42–1.97	.81
Feeding tubes (NT or PEG) (yes/no)	20/40	3.47	1.85–6.51	<.001	2.51	1.11–5.66	.03
Peripheral venous catheters (yes/no)	244/848	1.82	1.47–2.27	<.001	1.41	1.06–1.87	.02
Urinary catheters (yes/no)	199/539	2.79	2.23–3.50	<.001	1.73	1.30–2.29	<.001
Physical restraints (yes/no)	268/695	3.94	3.15–4.94	<.001	1.84	1.40–2.40	<.001

*OR* odds ratio, *95 % CI* 95 % confidence interval, *Ref* reference value for the hospital wards, *ADL* Activities of Daily Living score, *AChE-I* acetylcholinesterase inhibitors, *NT* nasogastric tube, *PEG* percutaneous endoscopic gastrostomy

## Discussion

To our knowledge, this is the first nationwide multicenter study to assess the prevalence of delirium in elderly patients across acute and rehabilitation hospital wards over a single day. Delirium prevalence was 22.9 % in the entire sample. The most common subtype was hypoactive, followed by mixed and then hyperactive and finally by nonmotoric. Prevalence was highest in Neurology and Geriatrics and lowest in Rehabilitation hospital wards. Delirium occurrence was independently associated with the more elderly, ADL dependence, dementia, malnutrition, and use of antipsychotics, feeding tubes, peripheral venous and urinary catheters, and physical restraints. Moreover, taking admission to Rehabilitation as a reference category, being admitted to Neurology wards was also associated with delirium occurrence, while admission to other settings was not.

Although our findings are consistent with those of the existing literature [4, 36], previous studies have assessed the prevalence of delirium in a single hospital [23–25, 37–39] or at no more than three hospitals [40, 41] and/or involved only one type of specialist ward per study (i.e., only Internal Medicine, Geriatrics, or Orthopedic units). Other studies conducted in Neurology and Orthopedic wards have mainly focused on the incidence of delirium rather than on its prevalence [4, 42–45], which explains the lack of such data in these settings. The few studies in Rehabilitation settings found a delirium prevalence ranging from 13–18 % [21, 22], but, again, these data referred to a single hospital or were combined. The results of the only point prevalence delirium study [26] available to date were similar to those of our study. Ryan and colleagues included 280 patients from different wards of a medium-size tertiary hospital in Ireland. Using a set of well-validated tools, a trained team of delirium experts found in this population a delirium prevalence of 19.6 % with the Diagnostic and Statistical Manual of Mental Disorders (DSM)-IV criteria, 17.6 % with the Confusion Assessment Method (CAM), and 20.7 % with the Delirium Rating Scale-Revised-98 (DRS-R98) [26]. Several hospital wards were screened for delirium prevalence, including Geriatrics and Internal Medicine, Oncology and Radiotherapy, General Surgery, Neurosurgery, and Orthopedics [26]. However, data from some wards were very limited in number, preventing the researchers from establishing a setting-specific prevalence of delirium.

In the present study, we achieved a large participation from Geriatric, Internal Medicine, Neurology, and Orthopedic wards distributed in 18 out of 20 Italian regions, allowing us to obtain a representative picture of the impact of delirium across Italian hospitals. These data confirm that delirium is not exclusive to specific healthcare settings, but is common in all studied hospital wards, in spite of the clear differences observed among patients in the different settings. The highest prevalence of delirium observed in Geriatric and Neurology wards might be explained by the highest prevalence of risk factors for delirium: old age, disability, and malnutrition, which were most frequent in Geriatrics, while both dementia and use of psychotropic medications were highest in Neurology wards. The independent association between the admission to Neurology wards and delirium might have several explanations. Consistent with patients’ features, the unmeasured confounding of behavioral and psychological symptoms associated with dementia might be more frequent in this setting. The presence of such symptoms might both increase the risk of delirium [45] and be misdiagnosed as delirium itself, especially for the features “alertness” and “fluctuating course” of the 4AT. Alternatively, a selection bias might be present, with delirium cases being referred more often to Neurology from emergency departments. Overall, these findings strongly suggest that delirium in acute Neurology wards should be regarded as a potential target of interest for future studies.

The other variables associated with delirium in the present study have all been recognized as predictors in previous prospective researches [4]. In particular, according to Inouye’s model [46], we found that both predisposing (i.e., age, disability, dementia, and malnutrition) and precipitating factors (i.e., use of antipsychotics, feeding tubes, peripheral venous and urinary catheters, and physical restraints) were associated with delirium occurrence in the whole population, thus confirming the multifactorial nature of this syndrome.

The frequency of the delirium motoric subtypes in our study is in keeping with previous ones carried out on smaller populations [47, 48]. Because the most prevalent delirium subtype was the hypoactive one, which is at highest risk of underdetection [28], we also claim the importance of an active case finding in clinical practice using standardized tools in order to avoid misdetection. Conversely, the hyperactive form of delirium, which is the most readily recognized, was also found to be the least prevalent.

The present study has several implications for clinicians and policymakers. Indeed, because delirium prevalence is in keeping with previous studies that used more accurate diagnostic methods [4, 26], our study indirectly shows that multicenter studies in this field are both feasible and reliable, even with physicians untrained in the diagnosis of delirium. We also pose that the use of 4AT in similar initiatives might be an aid in the comparison of delirium prevalence among different healthcare settings, increasing delirium awareness among healthcare providers. Finally, although a causal relationship could not be established in our study due to its cross-sectional design, the high prevalence of modifiable factors associated with delirium (malnutrition, antipsychotic use, feeding tubes, peripheral venous and urinary catheters, and physical restraints) suggests that a relationship may exist between delirium and these factors. Hence, future studies are needed to investigate potential relationships between these care practices and delirium.

Strengths of the present study include the large sample and the inclusion of more than 100 healthcare facilities from different settings. A second strength is the adoption of a common assessment protocol based on the detection of delirium with the 4AT, a simple tool that requires neither preliminary training nor longstanding experience to be administered. In fact, previous studies have generally used diagnostic instruments and/or criteria that are time-consuming or difficult to implement in clinical practice [27, 49], which may represent potential barriers to improving delirium detection in clinical practice [13, 50].

Some limitations of this study should be acknowledged. First, the participation in our study was on a volunteer basis, and therefore ours is a convenience sample. Second, it might be possible that centers participating in the present survey self-selected for a moderate-to-high attention to the issue and were therefore not fully representative of acute hospitals and rehabilitation settings in Italy. Third, we did not collect data regarding the main reasons of hospital admission, thus preventing us from assessing the role of precipitating factors of delirium, such as stroke and infections. Fourth, it might be possible that the diagnosis of dementia may have been underreported in medical records and that only a percentage of people with dementia would have been prescribed AChE-Is or memantine, leading to dementia being underreported in our study, and consequently, some cases of dementia may have been misclassified as delirium. Fifth, delirium in this study was defined with a brief assessment tool, the 4AT, rather than with a comprehensive reference standard. This was necessary for reasons of practicality; nevertheless, the tool has good sensitivity and specificity for delirium in the original study and in other published studies [51, 52]. Lastly, we cannot exclude the possibility that the 4AT may have at least partially overestimated the prevalence of delirium in subjects with pre-existing dementia. Indeed, in the validation study, the 4AT was shown to have good sensitivity but lower specificity in these patients [29].

## Conclusions

In conclusion, this is so far the largest point prevalence study of delirium on a national level, showing a prevalence of more than one out of five patients across different hospital wards. A reliable and widespread assessment of delirium may be obtained through the use of a simple and validated tool, thus allowing large-scale detection of this too often unrecognized condition. We suggest that “Delirium Day” might be performed in different countries and repeated over time in Italy, both as a supportive educational strategy to improve delirium awareness and as a potential benchmarking platform to assess the quality of assistance provided in different facilities.

## Abbreviations

AChE-I, acetylcholinesterase inhibitor; ADL, Activities of Daily Living; AIP, Italian Association of Psychogeriatrics; CAM, Confusion Assessment Method; DMSS, Delirium Motor Subtype Scale; DRS-R98, Delirium Rating Scale-Revised-98; DSM, Diagnostic and Statistical Manual of Mental Disorders; e-CRF, electronic case report form; IQR, interquartile range; NT, nasogastric tube; PEG, percutaneous endoscopic gastrostomy; SD, standard deviation; SIGG, Italian Society of Gerontology and Geriatrics; SIGOT, Italian Society of Hospital and Community Geriatrics; SINDEM Italian Society of Neurology for Dementia; SPSS, Statistical Package for the Social Sciences

## Additional file

Additional file 1: The 4AT test. (DOCX 51 kb)
