# Supplementary material for: “Delirium Day”: a nationwide point prevalence study of delirium in older hospitalized patients using an easy standardized diagnostic tool
Source: BMC Med. 2016 Jul 18;14:106. doi: 10.1186/s12916-016-0649-8 (PMC4950237; doi:10.1186/s12916-016-0649-8)
Supplement: Additional file 1: — The 4AT test. (DOCX 51 kb) [file 12916_2016_649_MOESM1_ESM.docx]

**Online supplemental materials**

**Appendix A: the 4AT**

**4AT**

**Assessment test**

**for delirium &**

**cognitive impairment**

*(label)*

**Patient name:**

**Date of birth:**

**Patient number:**

**_________________________________________________________**

**Date: Time:**

**Tester:**

**CIRCLE**

**[1] ALERTNESS**

*This includes patients who may be markedly drowsy (eg. difficult to rouse and/or obviously sleepy*

*during assessment) or agitated/hyperactive. Observe the patient. If asleep, attempt to wake with*

*speech or gentle touch on shoulder. Ask the patient to state their name and address to assist rating.*

Normal (fully alert, but not agitated, throughout assessment) **0**

Mild sleepiness for <10 seconds after waking, then normal **0**

Clearly abnormal **4**

**[2] AMT4**

*Age, date of birth, place (name of the hospital or building), current year.*

No mistakes **0**

1 mistake **1**

2 or more mistakes/untestable **2**

**[3] ATTENTION**

*Ask the patient: “Please tell me the months of the year in backwards order, starting at December.”*

*To assist initial understanding one prompt of “what is the month before December?” is permitted.*

Months of the year backwards Achieves 7 months or more correctly **0**

Starts but scores <7 months / refuses to start **1** Untestable (cannot start because unwell, drowsy, inattentive) **2**

**[4] ACUTE CHANGE OR FLUCTUATING COURSE**

*Evidence of significant change or fluctuation in: alertness, cognition, other mental function*

*(eg. paranoia, hallucinations) arising over the last 2 weeks and still evident in last 24hrs*

No **0**

Yes **4**

**4 or above:** possible delirium +/- cognitive impairment

**1-3:** possible cognitive impairment

**0:** delirium or severe cognitive impairment unlikely (but delirium still possible if [4] information incomplete)

**4AT SCORE**

**GUIDANCE NOTES** Version 1.2. Information and download: **www.the4AT.com**

The 4AT is a screening instrument designed for rapid initial assessment of delirium and cognitive impairment. A score of 4 or more *suggests* delirium but is not diagnostic: more detailed assessment of mental status may be required to reach a diagnosis. A score of 1-3 suggests cognitive impairment and more detailed cognitive testing and informant history-taking are required. A score of 0 does not definitively exclude delirium or cognitive impairment: more detailed testing may be required depending on the clinical context. Items 1-3 are rated *solely on* *observation of the patient at the time of assessment.* Item 4 requires information from one or more source(s), eg. your own knowledge of the patient, other staff who know the patient (eg. ward nurses), GP letter, case notes, carers. The tester should take account of communication difficulties (hearing impairment, dysphasia, lack of common language) when carrying out the test and interpreting the score.

**Alertness:** Altered level of alertness is very likely to be delirium in general hospital settings. If the patient shows significant altered alertness during the bedside assessment, score 4 for this item. **AMT4 (Abbreviated Mental Test - 4):** This score can be extracted from items in the AMT10 if the latter is done immediately before. **Acute Change or Fluctuating Course:** Fluctuation can occur without delirium in some cases of dementia, but marked fluctuation usually indicates delirium. To help elicit any hallucinations and/or paranoid thoughts ask the patient questions such as, “Are you concerned about anything going on here?”; “Do you feel frightened by anything or anyone?”; “Have you been seeing or hearing anything unusual?”

© 2011-2014 MacLullich, Ryan, Cash
